# Summer seasonal precipitation prediction in the middle and lower reaches of the Yangtze River using neural network approaches

**DOI:** 10.1371/journal.pone.0343910

**Published:** 2026-11-05

**Authors:** Xiran Ren, Lingya Li

**Affiliations:** APT MOBILE SATCOM LIMITED, Shenzhen, China; Texas A&M University, UNITED STATES OF AMERICA

## Abstract

Accurate prediction of summer precipitation is essential for agricultural planning and flood prevention. However, precipitation is strongly nonlinear. In the middle and lower reaches of the Yangtze River (MLYR), many interacting factors and large interannual variability make conventional forecasting methods unreliable. This study proposes neural network approaches to address this challenge. We select independent predictors from 130 circulation indices of the National Climate Center (NCC) and from the ERA5 monthly dataset, including sea surface temperature (SST), 500 hPa geopotential height, and land surface variables. Predictors are chosen through correlation, partial correlation, and autocorrelation analyses. They are then processed with detrended fluctuation analysis (DFA), standardization, and normalization. Four models are trained: a back propagation (BP) network, a gated recurrent unit (GRU) network, a coupled BP-GRU network, and a particle swarm optimization (PSO)-optimized coupled BP-GRU network. A strict chronological split is used (training: 1980–2000; validation: 2001–2010; testing: 2011–2024). Results are reported as the mean ± standard deviation over ten independent runs. The PSO-optimized coupled network performs best (test MSE = 0.0177 ± 0.0106; spatial correlation *r* = 0.44). It outperforms the standalone GRU (MSE = 0.0207 ± 0.0003), the coupled BP-GRU (MSE = 0.0357 ± 0.0175), and the standalone BP model (MSE = 0.0447 ± 0.0212). It is also the only network to beat the climatological baseline (MSE = 0.0181). The GRU is the most stable across runs but reproduces spatial patterns poorly. Overall, combining recurrent architectures with physical predictors through evolutionary optimization improves seasonal prediction. However, all models show limited skill (R^2^ near zero), reflecting the difficulty of this task. The approach offers a reference for flood-season forecasting in the MLYR, with potential use in agriculture and flood prevention.

## Introduction

Summer precipitation provides abundant water vapor resources, but it can also trigger meteorological disasters. China is located in the East Asian monsoon region, a high-risk area for heavy rains and floods. Studies indicate that from 1994 to 2013, meteorological disasters accounted for 55% of deaths and 87% of economic losses among all natural disasters in China; among these, precipitation-related hazards such as droughts, heavy rains, floods, and mudslides constituted the vast majority [1]. Therefore, accurate precipitation prediction is not only the foundation for guiding China’s agricultural development and maintaining the stable operation of relevant economic sectors, but also crucial for protecting people’s lives and property. One of the key regions during China’s annual flood season is the Lower-and-Middle Section of Yangtze River. This area is not only an economically developed region in China but also an important base for the country’s primary and secondary industries. Meanwhile, it is among the regions most severely affected by floods during each flood season, with a large number of affected people and a wide impact area every year. For example, the catastrophic floods in the summer of 1998 swept through multiple river basins including the Yangtze River, affecting 29 provinces (autonomous regions and municipalities directly under the Central Government) across the country. The affected area reached 2.12 million square kilometers, with 223 million people affected and over 4,000 deaths [2], exerting a severe impact on social production and people’s livelihood. The MLYR therefore combines high economic exposure with high hydrological risk. This situation is not unique to China. As global warming increases the variability of the water cycle, seasonal-scale precipitation forecasting has become a shared priority for climate-sensitive sectors worldwide, from agriculture and water resources to disaster management. Improving seasonal prediction in the MLYR thus carries national significance for China’s food and economic security, and broader relevance as a testbed for methods transferable to other monsoon regions. Seasonal-scale precipitation prediction has long been a key challenge in China’s meteorological field [3] and a complex global problem [4]. The practical problem is that without reliable seasonal outlooks, flood and drought preparedness decisions must be made with high uncertainty. Traditional precipitation prediction methods mainly include mathematical statistical methods, dynamical methods, and dynamical-statistical combined methods [5].

In terms of mathematical statistical methods: Liu T et al. [6] combined modified BP-CCA and ECC methods to establish an ensemble prediction model; Hu G et al. [7] conducted seasonal precipitation prediction using Empirical Orthogonal Function (EOF) decomposition and Canonical Correlation Analysis (CCA); Du L et al. [8] zoned China’s summer precipitation based on climatic characteristics and built a summer precipitation prediction model for China according to the zoning results.

As for physical statistical (dynamical) methods: Wu et al. [9] used NAO and ENSO indices as predictors to construct a seasonal prediction model for EASM precipitation; Fan Ke et al. [10] predicted summer precipitation in the Lower-and-Middle Section of Yangtze River using factors such as AAO; Benestad R E [11] explored a method based on the upper tail properties of precipitation extreme distribution functions, using an empirical-statistical downscaling physical statistical approach with an assumed physical upper bound to analyze extreme precipitation trends.

For dynamical-statistical combined methods: Hong Mei et al. [12] took EOF time coefficient sequences as variables of a dynamic statistical model and established a nonlinear dynamic statistical model for separated EOF time coefficients; Guo Qu et al. [13] found that the BCC-CSM downscaling prediction model can significantly improve the summer precipitation prediction ability in eastern China; Sun Chenghu et al. [14] proposed an optimal interpolation assimilation method that integrates dynamical and statistical prediction information, further enhancing the skill of dynamical-statistical combined methods in summer precipitation prediction;

Liu Ying et al. [15] developed the HSDP method to improve the dynamical-statistical combined prediction of summer precipitation in southern China; Gu Weizong et al. [16] conducted regional summer precipitation prediction (RSPP) based on downscaling methods; Schepen et al. [17] used a Bayesian model to combine observations with the correlation of lagged climate indices for seasonal-scale precipitation prediction in Australia, and the results showed that this model outperforms single dynamical or statistical prediction methods; Zheng Ran et al. [18] used the interannual increment method to model and simulate the annual growth of summer precipitation in southwest China. However, influenced by atmospheric circulation systems, thermal effects of local circulation, and topography, precipitation exhibits nonlinear dynamic characteristics across multiple time scales. Using only traditional methods to analyze and process meteorological data faces many difficulties [19], leading to low prediction accuracy. Especially for flood season precipitation prediction in the Lower-and-Middle Section of Yangtze River, due to numerous influencing factors, complex interaction processes, and significant interannual variability, traditional prediction methods cannot achieve accurate forecasts.

Neural network models can better address nonlinear problems and easily handle higher-resolution datasets. Neural networks use grid-like layered structures to perform nonlinear transformations on data, thereby extracting low-level features and abstracting high-level features. These highly abstracted high-level features are used for regression prediction of precipitation [20], helping to further improve the accuracy of summer precipitation prediction. Common neural networks applied in the meteorological field include BP (Back Propagation) neural networks [21,22].

Yet, BP neural networks have drawbacks such as low efficiency and high risk of training failure, making them increasingly inadequate for meteorological big data prediction research. Meteorological data are inherently time-series data. As a deep learning algorithm, RNN (Recurrent Neural Network) is particularly suitable for predicting and analyzing large-scale time-series data, and has achieved good prediction results in foreign meteorological fields [23–25]. On the other hand, using RNN alone without considering non-time-series predictors also results in weak model generalization ability and low prediction accuracy. Therefore, integrating the BP neural network and the GRU (Gated Recurrent Unit), this study proposes a coupled neural network model for seasonal-scale summer precipitation forecasting in the Lower-and-Middle Section of Yangtze River (pipeline shown in Fig 1).

**Fig 1:**
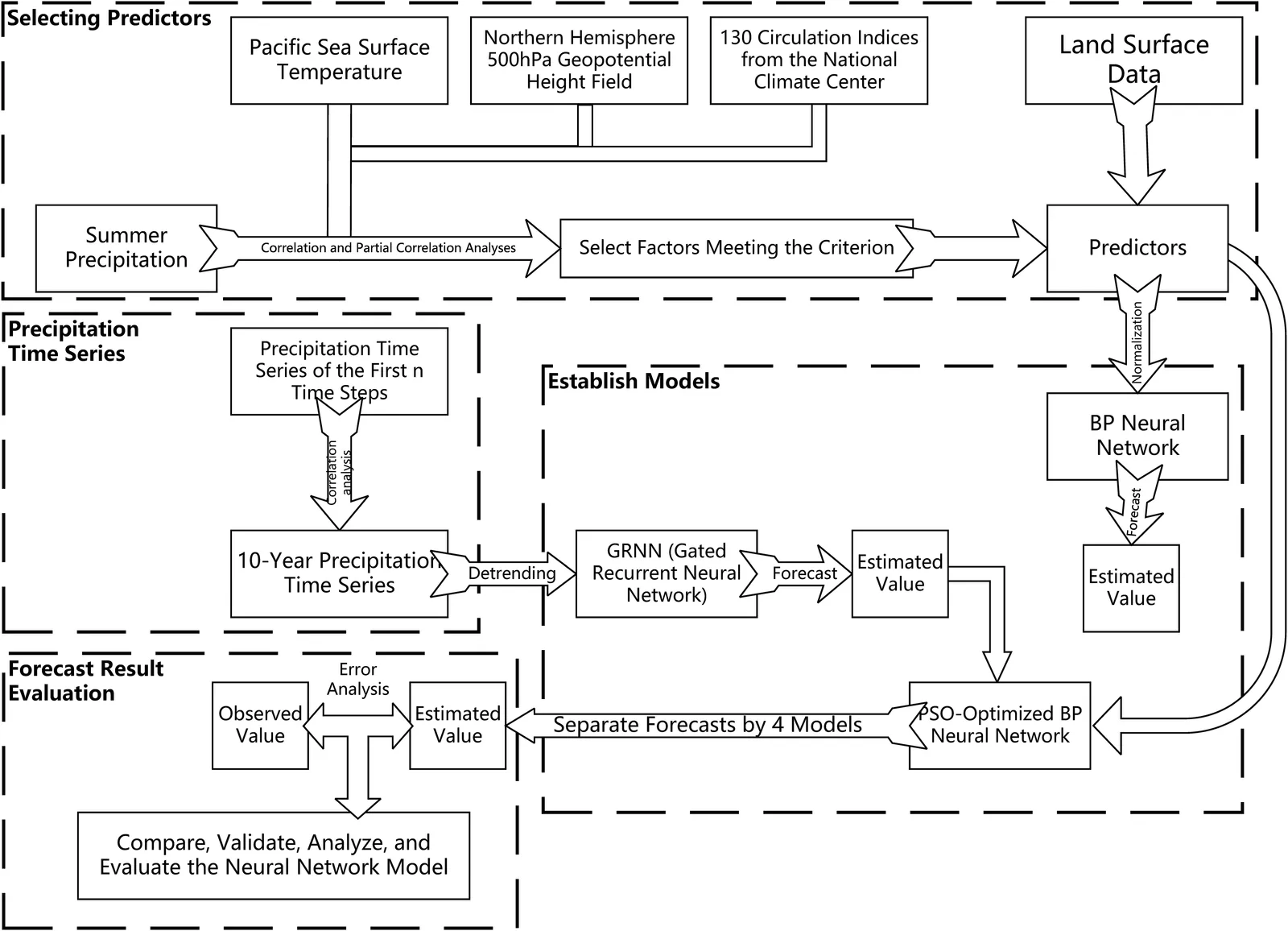
The pipeline of the research.

The coupled model is built on two complementary data sources: atmospheric and land-surface variables from ERA5 reanalysis and circulation indices from the National Climate Center (detailed in Section 2). From these, predictors significantly correlated with summer precipitation are selected via correlation, partial correlation, and autocorrelation analyses, then processed through detrended fluctuation analysis (DFA), standardization, and normalization.

The model adopts a “BP + GRU” coupling design: the GRU captures temporal dependencies in the precipitation time series, while the BP network learns nonlinear mappings from multiple physical predictors. The Particle Swarm Optimization (PSO) algorithm is further incorporated to optimize the weight matrices of the coupled network. Recent studies have demonstrated the potential of deep learning architectures—including CNN-LSTM hybrids [26] and comparative multi-model frameworks [27]—for climate prediction, motivating our exploration of coupled architectures for seasonal forecasting.

The overarching objective of this study is to develop a seasonal-scale model that produces usable summer precipitation outlooks for the MLYR, so that flood and drought preparedness decisions can be made earlier and with greater confidence. To this end, we pursue three specific aims. First, we identify a set of physically meaningful predictors from circulation indices, sea surface temperature, geopotential height, and land surface variables. Second, we construct and compare four neural network models that combine these predictors with the temporal structure of precipitation. Third, we evaluate all models under a strict chronological protocol, so that the reported skill reflects the true difficulty of the task rather than artifacts of random data splitting.

## Data and research area selection

### Research area selection and analysis

This study focuses on total summer (June–August) precipitation in the Lower-and-Middle Section of Yangtze River. The geographic scope of this region is defined as 27°N–34°N latitude and 111°E–123°E longitude [28], encompassing Jiangsu Province, Zhejiang Province, Hunan Province, Hubei Province, Jiangxi Province, Anhui Province, Fujian Province, Henan Province, and Shanghai Municipality. Fig 2 presents the 1979–2025 kernel density plot of monthly mean precipitation throughout the year in the Lower-and-Middle Section of Yangtze River. It can be observed that precipitation data in spring and winter are relatively concentrated, with the median in winter being the lowest; precipitation data in summer and autumn are more dispersed, and the median in summer is the highest. Fig 3 illustrates the spatial distribution of monthly mean precipitation during summer (June, July, August) in the Lower-and-Middle Section of Yangtze River. It can be seen that in June, a large-scale high-precipitation area exists at the border of Zhejiang and Jiangxi provinces, with a higher maximum precipitation than that recorded in July and August; in July, a distinct but smaller high-precipitation zone is located at the junction of Anhui and Hubei provinces; in August, the high-precipitation area in the region shifts southward to southern Zhejiang and the coastal areas of Fujian Province.

**Fig 2:**
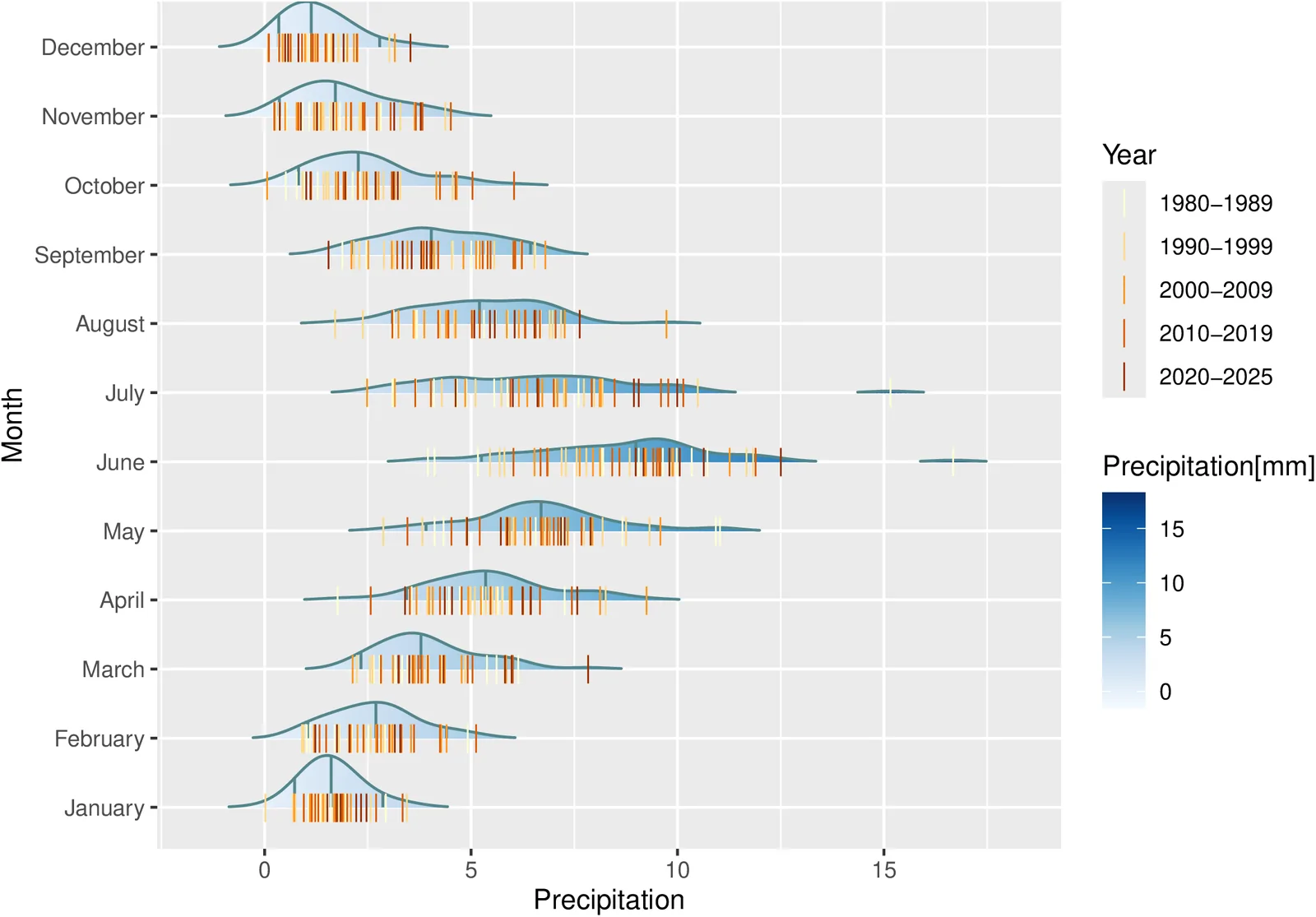
1979–2025 kernel density plot of monthly precipitation throughout the year in the lower-and-middle section of Yangtze River.

**Fig 3:**
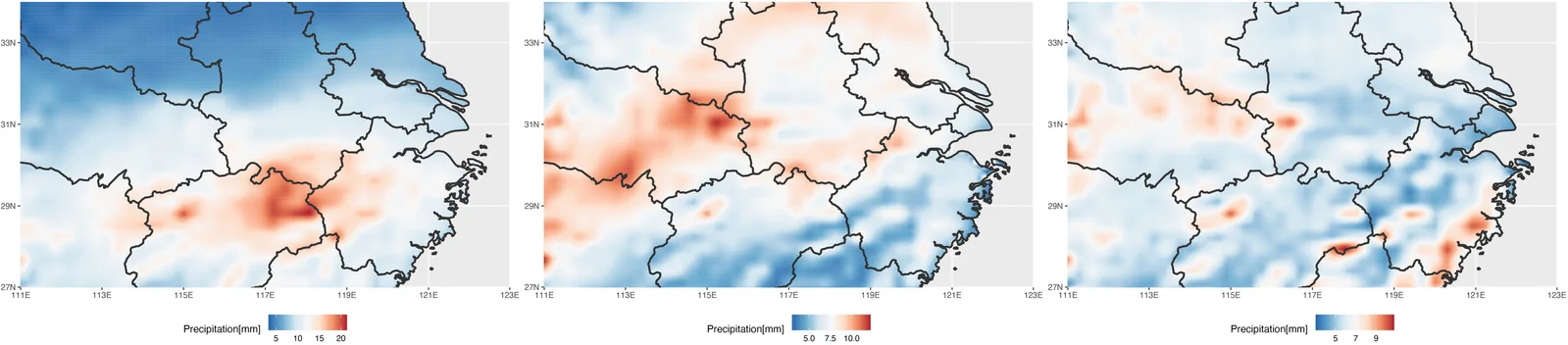
Spatial distribution of monthly mean precipitation during summer in the lower-and-middle section of Yangtze River.

### Data selection and analysis

These datasets are selected for model: (1) 130 circulation indices (88 atmospheric circulation indices, 26 sea surface temperature indices, and 16 other indices) from 1950 to 2025, provided by the National Climate Center, with a monthly temporal resolution. https://cmdp.ncc-cma.net/cn/monitoring.htm.

(2) ERA5 monthly averaged data on single levels(1979–present): Sea Surface Temperature (SST), more details are shown in Table 1. https://cds.climate.copernicus.eu/datasets/reanalysis-era5-single-levels-monthly-means?tab=overview.

**Table 1:** Overview of ERA5 monthly averaged data on single levels (sea surface temperature).

Property	Value
Variable	Sea surface temperature (SST)
Unit	K
Description	Temperature of sea water near the surface; a foundation SST with no diurnal variations
Data type	Gridded
Projection	lat/lon grid
Range	75°W–105°E, 66.5°S–66.5°N
Temporal resolution	Monthly
Time period	1979–2025

(3) ERA5 monthly averaged data on pressure levels (1979–present): 500hPa Geopotential Height, more details are shown in Table 2. https://cds.climate.copernicus.eu/datasets/reanalysis-era5-pressure-levels-monthly-means?tab=overview.

**Table 2:** Overview of ERA5 monthly averaged data on pressure levels (500 hPa geopotential height).

Property	Value
Variable	500 hPa geopotential height
Unit	m^2^s^–2^
Description	Gravitational potential energy of a unit mass at a particular location relative to mean sea level
Data type	Gridded
Projection	lat/lon grid
Range	180°W–180°E, 0°–90°N
Temporal resolution	Monthly
Time period	1979–2025

(4) ERA5-Land monthly averaged data (1979–present): 10m u-component of wind, 10m v-component of wind, 2m dewpoint temperature, 2m temperature, surface pressure and total precipitation, more details are shown in Table 3. https://cds.climate.copernicus.eu/datasets/reanalysis-era5-land-monthly-means?tab=overview

**Table 3:** Overview of ERA5-Land monthly averaged data (10m u-component of wind, 10m v-component of wind, 2m dewpoint temperature, 2m temperature, surface pressure and total precipitation).

Variable	Unit	Description
10m u-component of wind	ms^–1^	Eastward component of the 10m wind (horizontal speed of air moving toward the east at 10 m above the surface)
10m v-component of wind	ms^–1^	Northward component of the 10m wind (horizontal speed of air moving toward the north at 10 m above the surface)
2m dewpoint temperature	K	Temperature to which the air at 2 m above the surface would have to be cooled for saturation to occur
2m temperature	K	Temperature of air at 2 m above the surface of land, sea or inland waters
Surface pressure	Pa	Pressure (force per unit area) of the atmosphere at the surface of land, sea or inland water
Total precipitation	m	Accumulated liquid and frozen water (rain and snow) that falls to the surface

These four datasets were selected because they jointly capture the two classes of information needed for seasonal prediction. The 130 circulation indices summarize the large-scale atmospheric and oceanic modes that, on the basis of dynamical theory, are known to drive East Asian summer monsoon variability. The ERA5 single-level, pressure-level, and land variables provide the spatially resolved sea surface temperature, mid-tropospheric circulation, and near-surface thermodynamic and wind fields from which localized predictors can be extracted. ERA5 was chosen over alternative reanalyses because it offers global coverage, a consistent assimilation system, and monthly means at a resolution suitable for regional predictor screening. All ERA5 variables are available from 1979, the start of the modern satellite era, when the volume of assimilated observations increases sharply and the reanalysis becomes homogeneous in quality. We therefore restrict the entire analysis to the common period 1979–2025. This 46-year record is long enough to capture several decades of interannual variability, while remaining short enough to keep the predictor-to-sample-size ratio reasonable for a machine-learning model. The circulation indices, although available from 1950, are used only over 1979–2025 to align with ERA5.

#### SST, 500hPa geopotential height and circulation indices data analysis.

Surveys show that summer precipitation in eastern China exhibits significant intraseasonal oscillations (ISO) [29]. Such oscillations regulate the duration and progression of the rainy season in eastern China, with the Lower-and-Middle Section of Yangtze River and its southern regions dominated by ISOs with a 30–60-day period [30]. Wang et al. [31] investigated seasonal-scale air-sea interactions in the Kuroshio Extension region and found that local air-sea interactions may contribute to these ISOs in the target area. Thus, this study selects Pacific sea surface temperature as one of the precipitation predictors. The 500hPa layer is approximately a region of non-convergence and non-divergence, making it relatively stable with distinct ascending and descending zones. Monthly mean 500hPa geopotential height can derive variable fields such as vorticity changes, meridional wind shear, and zonal wind shear [32] for precipitation forecasting. Synoptic analysis also frequently relies on 500hPa weather charts to study precipitation. Additionally, mature ensemble forecast products for monthly mean 500hPa geopotential height enable reliable predictive performance [33]. Therefore, this study adopts the Northern Hemisphere 500hPa geopotential height as another key predictor. Short-to-medium-term variations in atmospheric circulation are closely linked to the formation, development, and dissipation of weather systems, thereby profoundly influencing regional synoptic conditions and precipitation [34]. Circulation indices are commonly used in meteorology to characterize the alternating evolution of upper and lower atmospheric systems. Accordingly, this study chooses 130 circulation indices from the National Climate Center as a third set of precipitation predictors.

#### Dewpoint temperature, surface pressure, temperature, total precipitation, u-component of wind and v-component of wind speed data analysis.

Fig 4 presents the correlation coefficients between monthly mean 2m dewpoint temperature, 2m temperature, surface pressure, 10m u-component of wind and v-component of wind speed (1979–2025) and monthly mean summer precipitation (June–August) in the Lower-and-Middle Section of Yangtze River, along with bivariate distribution maps of the monthly mean precipitation for the same period. Notably, June precipitation maxima exhibit a strong positive correlation with 2m dewpoint temperature, significant areas are primarily concentrated in the northern part of the basin. In July, a large region of strong positive correlation spans 27°N–28°N, with significant areas scattered across both southern and northern parts of the basin. August shows weaker correlation coefficients overall, but the extent of statistically significant areas is larger than that in July. Surface pressure displays substantial correlation with monthly mean precipitation across June–August. In June, correlation coefficients are relatively weak, yet significant areas cover nearly the entire north-central basin. Extensive strong negative correlation zones linked to heavy precipitation, mainly in the northern basin in July. In August, the northern region shifts to strong positive correlation, with strong negative correlation zones moving southward. Significant areas are widespread for the correlation between 2m temperature and precipitation. June sees a large region of positive correlation in the central basin, almost all of which passes the significance test. In July and August, the entire basin is significant, with small patches of strong positive correlation in the south. Correlation analysis between monthly mean precipitation and monthly mean 10m u-component of wind speed reveals the following patterns: in June, a small patch of strong positive correlation (associated with heavy precipitation) lies at the junction of Anhui, Jiangxi, and Zhejiang provinces. In August, there is a large region of positive correlation corresponding to relatively high monthly average precipitation. Correlation analysis between monthly mean precipitation and monthly mean 10m v-component of wind speed yields the following findings: in August, large swathes of strong negative correlation span the eastern area, interspersed with discontinuous precipitation maxima. In summary, monthly mean 2m dewpoint temperature, 2m temperature, surface pressure, u-component of wind and v-component of wind speed all exhibit meaningful correlations with summer (June–August) precipitation in the study area. Thus, these variables are selected as predictors in this study.

**Fig 4:**
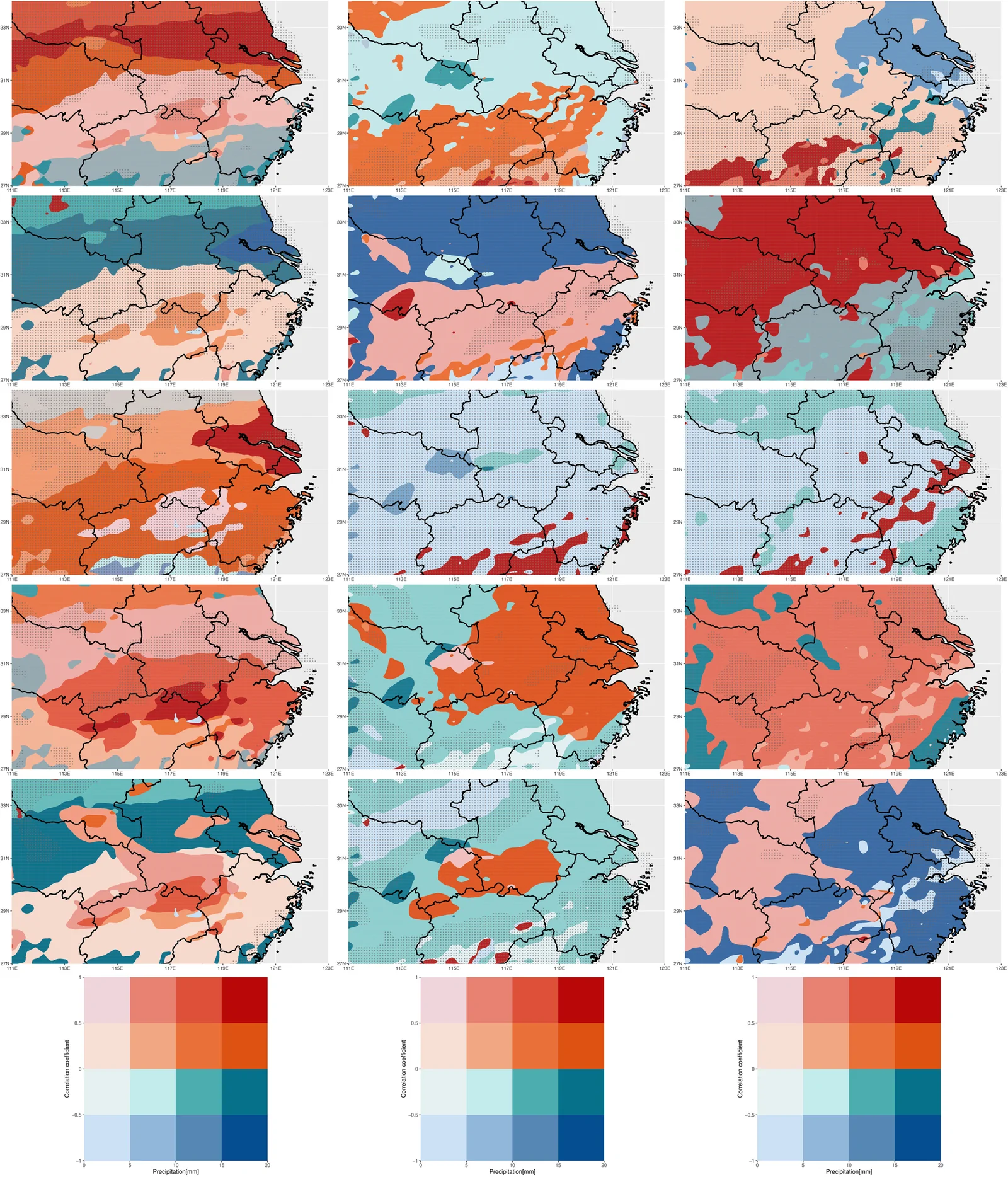
Spatial distribution of correlation coefficients between monthly mean 2m dewpoint temperature, 2m temperature, surface pressure, 10m u-component of wind and v-component of wind speed (1979–2025) and monthly mean summer precipitation (June–August) in the Lower-and-Middle Section of Yangtze River, along with spatial distribution of the monthly mean precipitation (shaded areas indicate statistical significance at the 90% confidence level).

#### Time series prediction factors analysis.

Precipitation time series exhibit inherent autocorrelation, enabling the use of precipitation data from the previous n time steps (here, n = 10, meaning precipitation from the prior 10 years is chosen as predictors) to forecast the current precipitation time series. As illustrated in Fig 5, the previous decade’s precipitation shows a meaningful linear correlation with current data, with similar density distributions. Additionally, summer precipitation is higher and more dispersed than in other seasons. The maximum monthly mean summer precipitation occurred during the 1980s.

**Fig 5:**
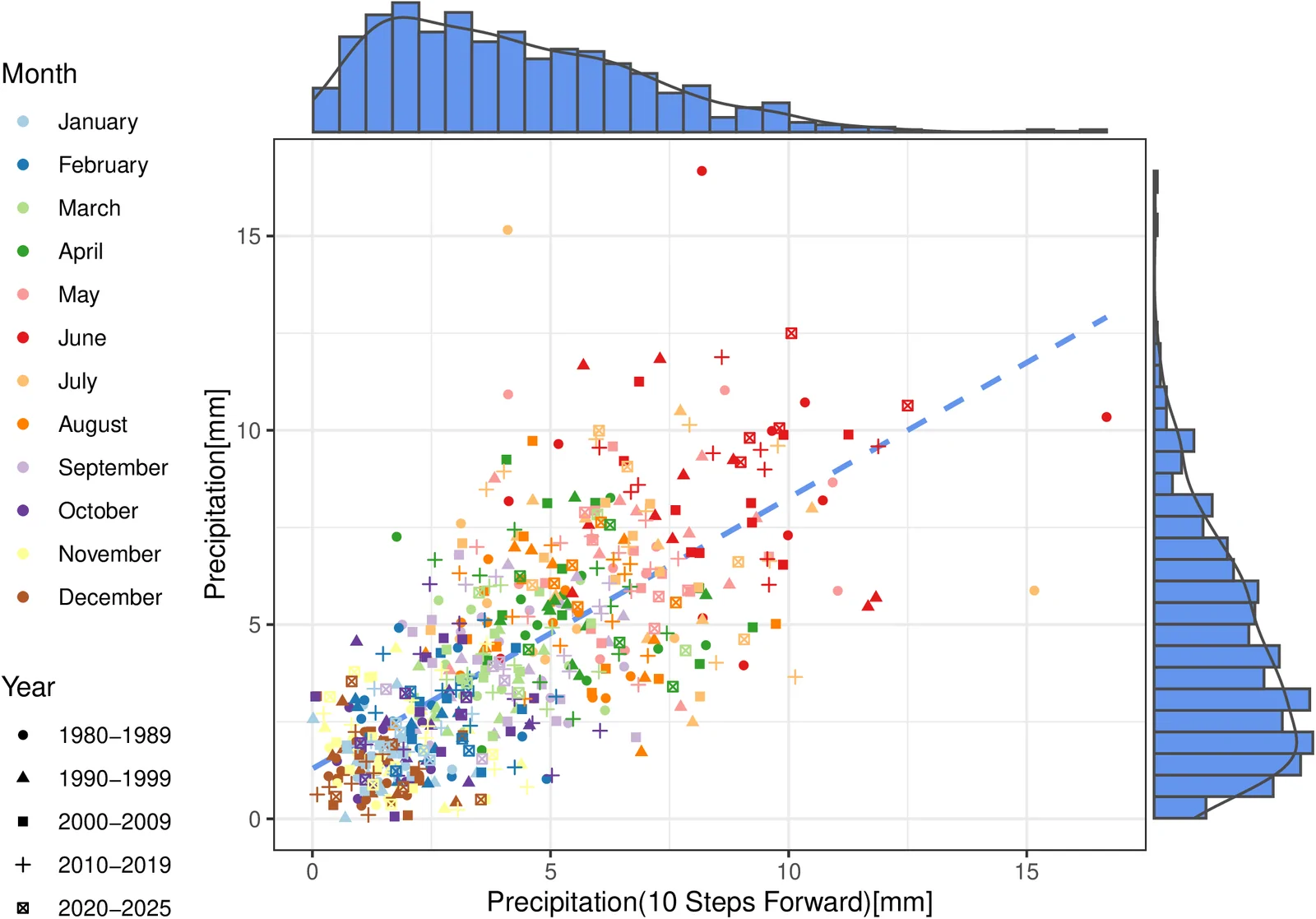
Scatter plot of the current precipitation and the precipitation for the preceding 10 years.

To further validate the choice of a 10-year lag window, we conduct autocorrelation function (ACF) and partial autocorrelation function (PACF) analyses on the detrended summer precipitation time series (Fig 6). The ACF quantifies the linear correlation between precipitation at time *t* and its value at lag *k*, while the PACF measures the same correlation after removing the effects of intermediate lags. The ACF decays gradually from approximately 0.6 at lag 1 to non-significant values beyond lag 10, indicating that precipitation autocorrelation persists for roughly one decade. The PACF shows significant partial autocorrelation at lags 1–3, with values approaching zero thereafter, suggesting that the direct dependence structure is concentrated within the first few years.

**Fig 6:**
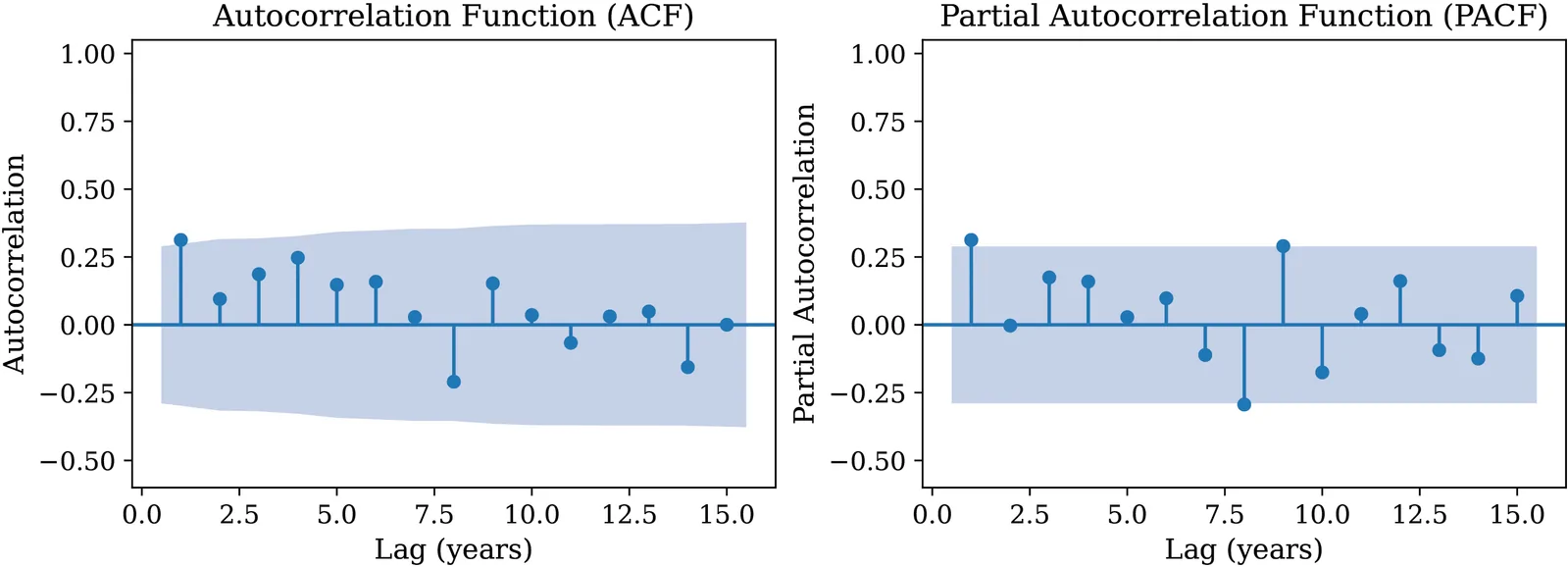
ACF and PACF of the detrended summer precipitation time series (1979–2025). Dashed lines indicate the 95% confidence bounds ($\pm 1.96/\sqrt{n}$).

The combination of the sustained ACF decay up to lag 10 and the PACF cutoff at shorter lags provides quantitative justification for selecting precipitation from the preceding 10 years as predictors: this window is long enough to capture the full autocorrelation structure while short enough to maintain a reasonable predictor-to-sample-size ratio for a 46-year record. This study selects a composite of precipitation time series from the previous 1–10 years relative to the current period as key predictors.

## Method

### Predictor selection

A central step in seasonal prediction is selecting a small set of predictors from a large candidate pool. This selection is guided by the physical principle of lagged teleconnection: slowly varying boundary forcings, such as sea surface temperature anomalies and persistent circulation regimes, influence summer precipitation through atmospheric teleconnections that operate over weeks to seasons. Predictors identified in this way are physically meaningful rather than statistical artifacts. We therefore quantify the linear relationship between each candidate predictor in the preceding autumn, winter, and spring, and the following summer precipitation, using the Pearson correlation coefficient, and retain only those with a statistically significant and sufficiently strong association. Partial correlation is used to remove the influence of a third variable, and autocorrelation analysis is used to capture the temporal persistence of precipitation itself. Together these three analyses serve the first research aim of identifying an independent, physically grounded predictor set, and they reduce dimensionality so that the neural networks can be trained on a 46-year record without overfitting.

### BP neural network

The learning process of a BP neural network consists of two stages: forward propagation of data and backward propagation of error [35]. During the forward propagation process, the network weights a series of data inputs to control the proportion each input contributes, then feeds these weighted inputs to the hidden layer. Each neuron in the hidden layer sums all its inputs, applies an activation function to produce output, and then feeds this weighted output to the output layer. Each neuron in the output layer sums all its inputs to generate the final output. During backward propagation process, the actual output is compared with the desired output to calculate the error. This error is then used to adjust the connection weights between layers and the parameters of each neuron. The training process repeats these steps cyclically until the error falls below a predefined threshold.

### Recurrent neural networks

Recurrent Neural Networks (RNNs) are characterized by their recurrent structure, where connections can be established both within individual neurons and between neurons. This allows RNNs to store information from previous time steps and influence the output of subsequent steps, thereby enabling more effective processing of time-series data. The hidden layer of the GRU recurrent neural network is composed of several GRU blocks connected in the time dimension, and each GRU block is composed of a reset gate and an update gate. The GRU block calculation formula is as follows:


$$\begin{array}{c} \left\{ \begin{array}{c} r_{t}=\sigma \left(W_{r}*\left[h_{t-1},x_{t}\right]+b_{r}\right)\\ z_{t}=\sigma \left(W_{z}*\left[h_{t-1},x_{t}\right]+b_{z}\right)\\ {\tilde{h}}_{t}=\tanh\left(x_{t}W_{hx}+r_{t}⊙h_{t-1}W_{hh}+b_{h}\right)\\ h_{t}=\left(1-z_{t}\right)⊙h_{t-1}+z_{t}{\tilde{h}}_{t} \end{array}\right. \end{array}$$ (1)

where r_t_ is the reset gate, z_t_ is the update gate, x_t_ is the input at time t, h_t_ is the output of the hidden layer GRU block at time t, ⊙ is element-wise multiplication, σ is the Sigmoid function, and W and b are both trainable parameters of the hidden layer.

### Particle Swarm optimization

Eberhart and Kennedy [36] proposed the particle swarm optimization algorithm (PSO) and constructed a particle swarm to simulate the behavior strategy of a flock of birds in foraging and path-finding, using this strategy to solve the minimum problem. We use the PSO algorithm to optimize our model. The steps are as follows.

(1) Set the fitness function to mean square error (MSE), and the fitness of each particle is calculated by the fitness function ($f\left(W_{ij},W_{jt}\right)$). The purpose of this method is to find the neural network weight matrix corresponding to the minimum MSE.

$$\begin{array}{c} \min f\left(W_{ij},W_{jt}\right)=\frac{\sqrt{\frac{1}{n_{1}}\sum_{i=1}^{n_{1}}\left(y\_train(i)-\hat{y}\_train(i)\right)}+\sqrt{\frac{1}{n_{2}}\sum_{i=1}^{n_{2}}\left(y\_test(i)-\hat{y}\_test(i)\right)}}{2} \end{array}$$ (2)

(2) Initially, the population iteration number M, population size N (i.e., the number of particles), learning factors c_1_, c_2_, inertia weight w are manually set, and the position x of the population is initialized, and the fitness function is used to calculate the initial fitness of N particles.
(3) Start iteration, update the particle’s position x and velocity v, and limit the position and velocity to fixed upper and lower bounds:

$$\begin{array}{c} \begin{array}{c} v\left(m\right)=w*v\left(m-1\right)+c_{1}*r_{1}\left(pbest\left(m\right)-x\left(m\right)\right)+c_{2}*r_{2}\left(pbest\left(m\right)-x\left(m\right)\right)\\ x\left(m\right)=x\left(m-1\right)+v\left(m-1\right)*t \end{array} \end{array}$$ (3)

where m is the current number of iterations, r_1_ and r_2_ are random numbers of (0,1), and w is the inertia weight used to maintain the speed influence of the particle in the previous iteration.
(4) Input x as a weight matrix to the model for update, calculate the fitness of each of the N particles after the update, and update the local optimal position pbest of the current particle n.

$$\begin{array}{c} \left\{ \begin{array}{c} pbest\left(m,n\right)=x\left(m,n\right)\;\;,pbest\left(m-1,n\right)>x\left(m,n\right)\\ pbest\left(m,n\right)=pbest\left(m-1,n\right)\;\;,pbest\left(m-1,n\right)⩽x\left(m,n\right) \end{array}\right. \end{array}$$ (4)

(5) Update the global optimal particle position gbest:

$$\begin{array}{c} \left\{ \begin{array}{c} gbest\left(m\right)=pbest\left(m\right)\;\;,gbest\left(m-1\right)>pbest\left(m\right)\;\;\\ gbest\left(m\right)=gbest\left(m-1\right)\;\;,gbest\left(m-1\right)⩽pbest\left(m\right)\;\; \end{array}\right. \end{array}$$ (5)

(6) Perform steps 3–5 in a loop until the cycle number M is reached, and output the optimized model.

### Detrended fluctuation analysis

Detrended fluctuation analysis can eliminate linear or nonlinear trend components in the data to eliminate their interference on the result analysis. For a given time series *x*(*t*) (*t* = 1,2,3...*N*), the calculation steps of the DFA method [37] are as follows:

(1) Calculation of cumulative dispersion of *x*(*t*) (*t* = 1,2,3...*N*), where $\overset{―}{x}=\frac{1}{N}\sum_{t=1}^{N}x\left(t\right)$:

$$\begin{array}{c} Y\left(i\right)=\sum_{t=1}^{i}\left(x\left(t\right)-\overset{―}{x}\right)\;\;,i=1,2,3...N \end{array}$$ (6)

(2) Divide Y(i) into intervals of length s, where $N_{s}=int\left(\frac{N}{s}\right)$. Because the sequence length is not necessarily an integer multiple of s, Y(i) is divided twice from large to small and from small to large to avoid losing the tail information, thus obtaining $2N_{s}$ sub-intervals.
(3) For s points in each interval v (v = 1,2,3...2), using polynomial fitting of the least squares method, the fitting equation can be obtained as follows:

$$\begin{array}{c} y_{v}\left(i\right)=a_{0}+a_{1}i+a_{2}i^{2}+...+a_{k}i^{k}\;\;,i=1,2...,s,k=1,2,... \end{array}$$ (7)

as $\max\left\{k+2,10\right\}⩽s⩽\frac{N}{4}$.
(4) Calculate the mean square error $F^{2}\left(v,s\right)$. When $v=1,2,3...,N_{s}$,

$$\begin{array}{c} F^{2}\left(v,s\right)=\frac{1}{s}\sum_{i=1}^{s}{\left\{Y\left[\left(v-1\right)s+i\right]-y_{v}\left(i\right)\right\}}^{2} \end{array}$$ (8)

When $v=N_{s}+1,N_{s}+2,N_{s}+3...,2N_{s}$,

$$\begin{array}{c} F^{2}\left(v,s\right)=\frac{1}{s}\sum_{i=1}^{s}{\left\{Y\left[N-\left(v-N_{s}\right)s+i\right]-y_{v}\left(i\right)\right\}}^{2} \end{array}$$ (9)

(5) For interval $2N_{s}$, use the mean $F^{2}\left(v,s\right)$ to calculate the fluctuation function *F*(*s*):

$$\begin{array}{c} F\left(s\right)={\left\{\frac{1}{2N_{s}}\sum_{v=1}^{2N_{s}}\left[F^{2}\left(v,s\right)\right]\right\}}^{\frac{1}{2}} \end{array}$$ (10)

(6) When the time series *x*(*t*) has long-range correlation, *F*(*s*) and s satisfy the relationship

$$\begin{array}{c} F\left(s\right)\;s^{h} \end{array}$$ (11)

For each s, the corresponding *F*(*s*) can be found. After fitting using the least squares method, the relationship between the slope value h and the Hurst index H can be obtained as: (a) If the sequence is a non-stationary sequence, then H=h−1; (b) If the sequence is a stationary sequence, then H = h.

### Normalization and standardization

In the neural network learning process, if the gradient descent algorithm is used to solve the problem, normalization and standardization can speed up the solution of the model. And when the dimensions of the independent input variables x are inconsistent, the meaning of the regression coefficient may be wrong, so it is necessary to process all the variables x into the same dimension. The standardized formula is $X_{i}=\frac{X_{i}-\mu }{\sigma }$, where μ is the mean of the data and σ is the standard deviation of the data. And the normalized formula is $X_{i}=\frac{X_{i}-X_{\min}}{X_{\max}-X_{\min}}$.

## Experimental setup and predictor selection

### Predictor processing

#### Selection of SST, 500hPa geopotential height and circulation indices.

We first perform spatial averaging on ERA5 monthly mean precipitation grid data, and sum the values for June, July, and August to generate the summer precipitation time series. We then conduct correlation analyses between this series and Pacific sea surface temperature (SST) data from the preceding autumn, preceding winter, and current spring, respectively. Fig 7 depicts the spatial distribution of correlation coefficients between summer precipitation in the Lower-and-Middle Section of Yangtze River and SST in other three seasons. It can be observed that regions with strong correlations between preceding autumn SST and summer precipitation are mainly concentrated in the central Pacific, showing strong negative correlation. Compared with preceding autumn, the preceding winter sees less correlation zones. The strong negative correlation zones still locate in eastern part of Southeast Asia. In spring, the correlation becomes weaker. In this study, we choose SST regions with absolute correlation coefficients |r|⩾0.595 that pass the 90% significance test as predictors for summer precipitation in the target basin. Specifically, data corresponding to 12 grid points of spring SST are adopted as predictors, denoted as S1-12.

**Fig 7:**
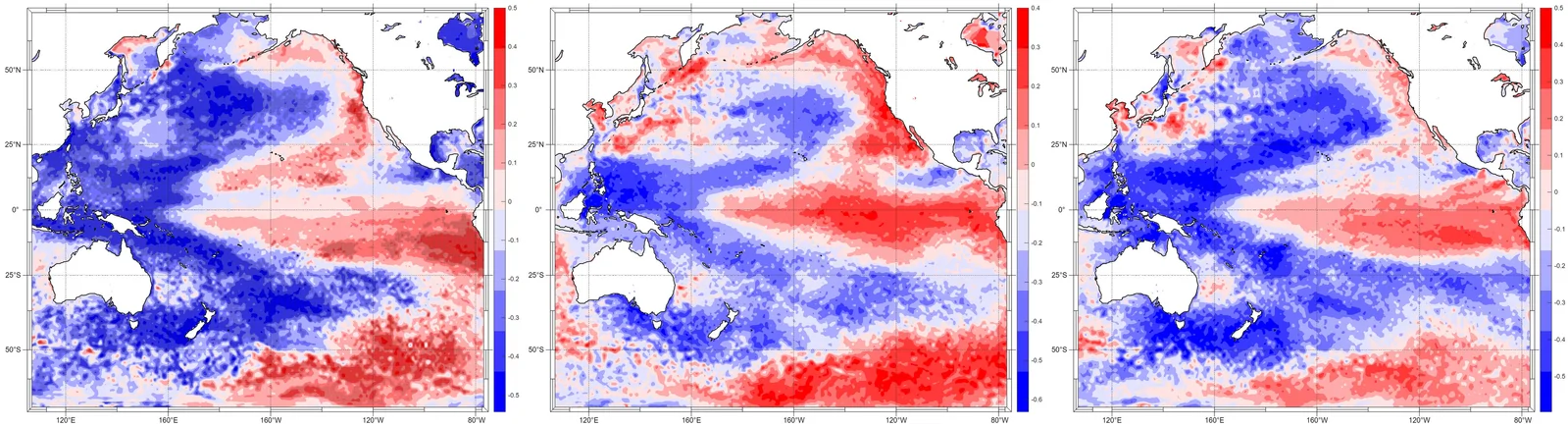
Spatial distribution of correlation coefficients between summer precipitation in the lower-and-middle section of Yangtze River and SST in other three seasons (shaded areas indicate statistical significance at the 90% confidence level).

The same summer precipitation time series (processed via the above method) is used for correlation analysis with the Northern Hemisphere 500hPa geopotential height field. Fig 8 presents the spatial distribution of correlation coefficients between summer precipitation in the Lower-and-Middle Section of Yangtze River and the 500hPa geopotential height field. Negative correlation dominates the preceding autumn and winter, with extensive strong negative correlation zones in Siberia and Eastern Europe. Correlation coefficient patterns in spring are more complex: strong positive correlation zones are primarily distributed across Russian, while strong negative correlation zones are located in the Midwestern USA, the Middle East, Egypt, India, and areas adjacent to the Qinghai-Xizang Plateau of China. We choose regions with absolute correlation coefficients |r|⩾0.547 that pass the 90% significance test. Specifically, data corresponding to 16 grid points of the preceding winter Northern Hemisphere 500hPa geopotential height field are chosen as predictors, denoted as H1-16. Correlation coefficient is calculated with the current summer precipitation from 1980 to 2025 and the circulation indices in January, February, March, April, and May of the corresponding year, in September, October, November, and December of the previous year. For the circulation indices that satisfy |r|⩾0.5 and pass the 90% significance test are selected as predictors. Finally, 11 predictors I1-11 are selected from 130 circulation indices.

**Fig 8:**
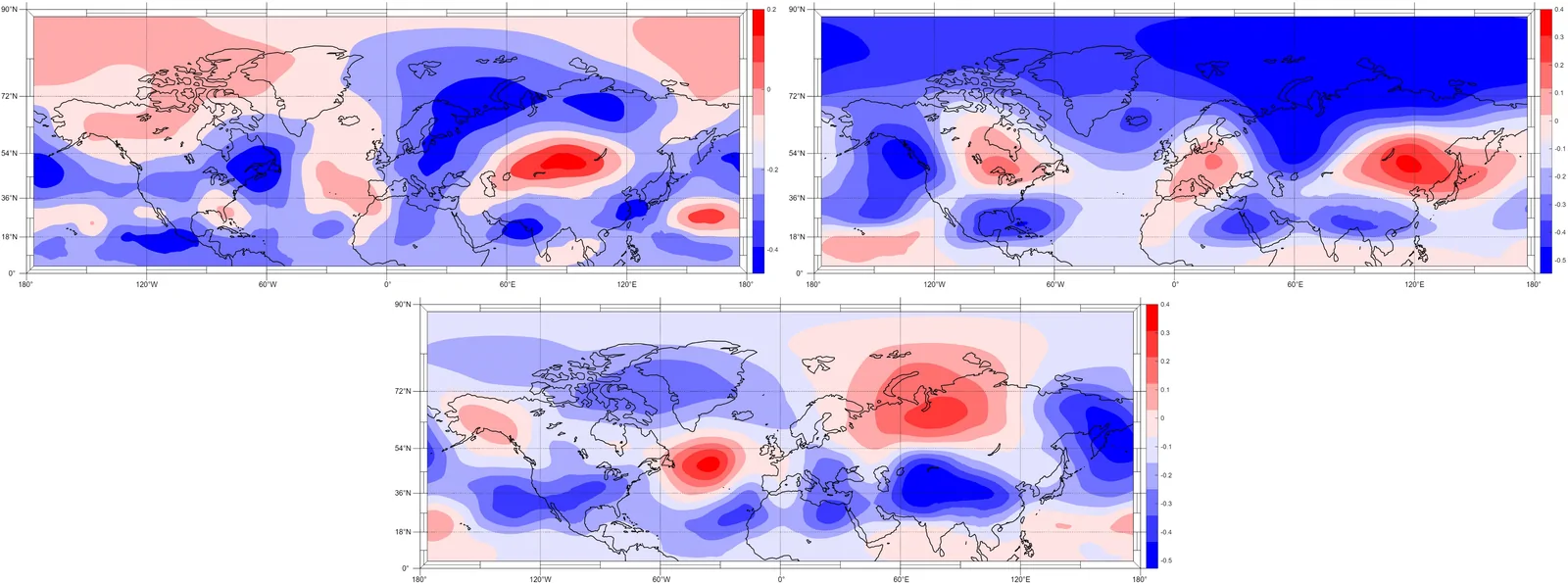
Spatial distribution of correlation coefficients between summer precipitation in the lower-and-middle section of Yangtze River and 500hPa geopotential height in other three seasons (shaded areas indicate statistical significance at the 90% confidence level).

#### Intercorrelation analysis among predictors.

As shown in Fig 9, before removing highly intercorrelated predictors, strong pairwise correlations exist among the 16 geopotential height predictors and among the 12 SST predictors, respectively. After eliminating predictors with strong pairwise correlations, we retain 16 predictors in total: monthly mean 2m dewpoint temperature, 2m temperature, surface pressure, 10m u-component of wind and v-component of wind speed, I1, I2, I4, I6, I7, I9, I10, I11, S1, S2 and H1. As illustrated in Fig 9(b), the pairwise correlation coefficients among these remaining 16 predictors are all relatively low.

**Fig 9:**
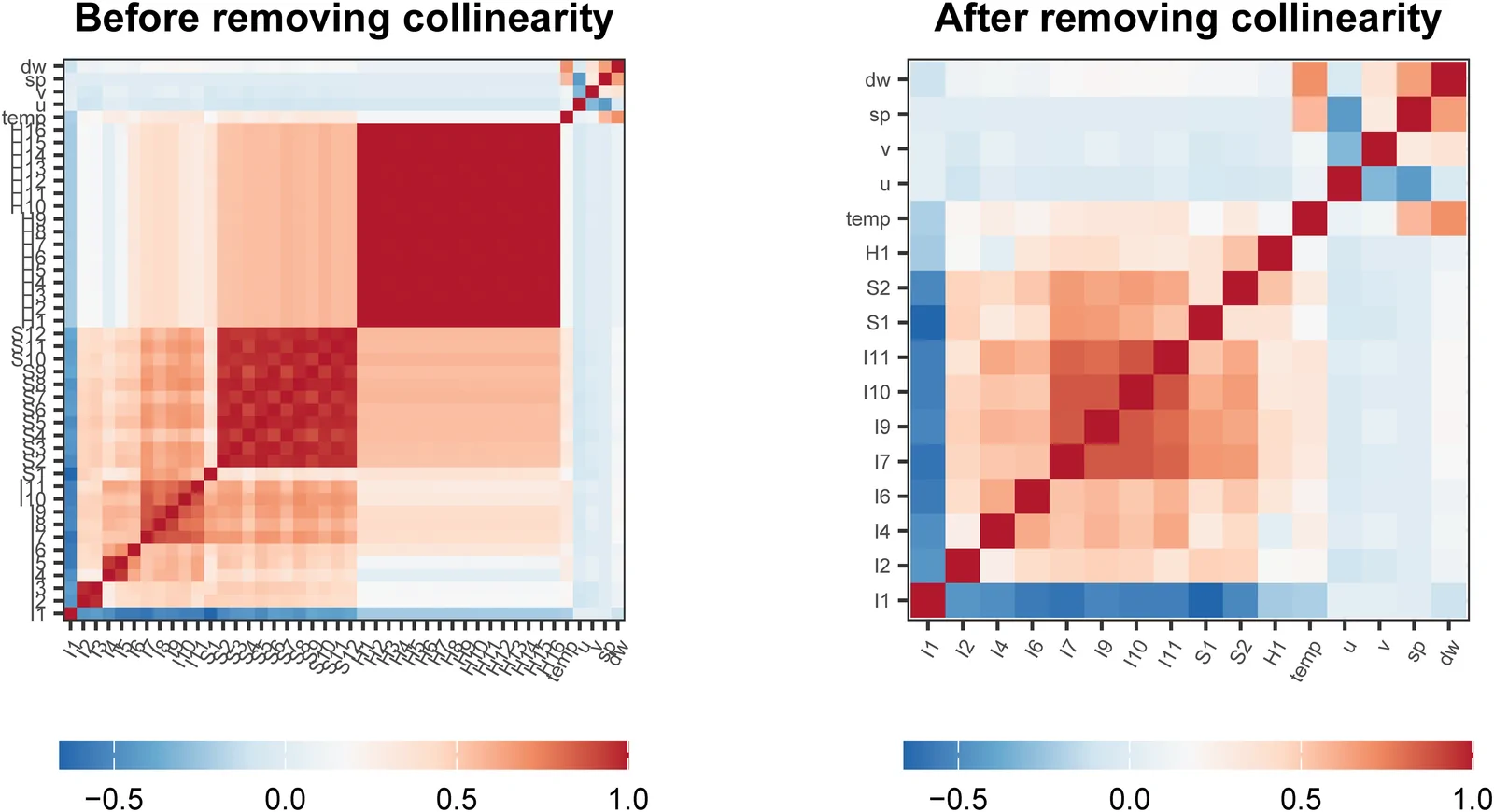
Predictor partial correlation coefficient heatmap.

#### Prediction time series detrending.

The climate system is not governed by a single meteorological factor; the vast majority of its signals are non-stationary time series, meaning that its mean, variance, autocorrelation function and other statistical properties vary over time. We introduce the DFA method to stationarize the signals of the climate system and extract the long-range correlation of precipitation time series, which we then use as input to the model. As shown in Fig 10, the detrended data eliminate the trend of the original dataset (blue line in the figure), while retaining certain fluctuation characteristics of the raw data, thus effectively extracting the periodic properties of the precipitation series.

**Fig 10:**
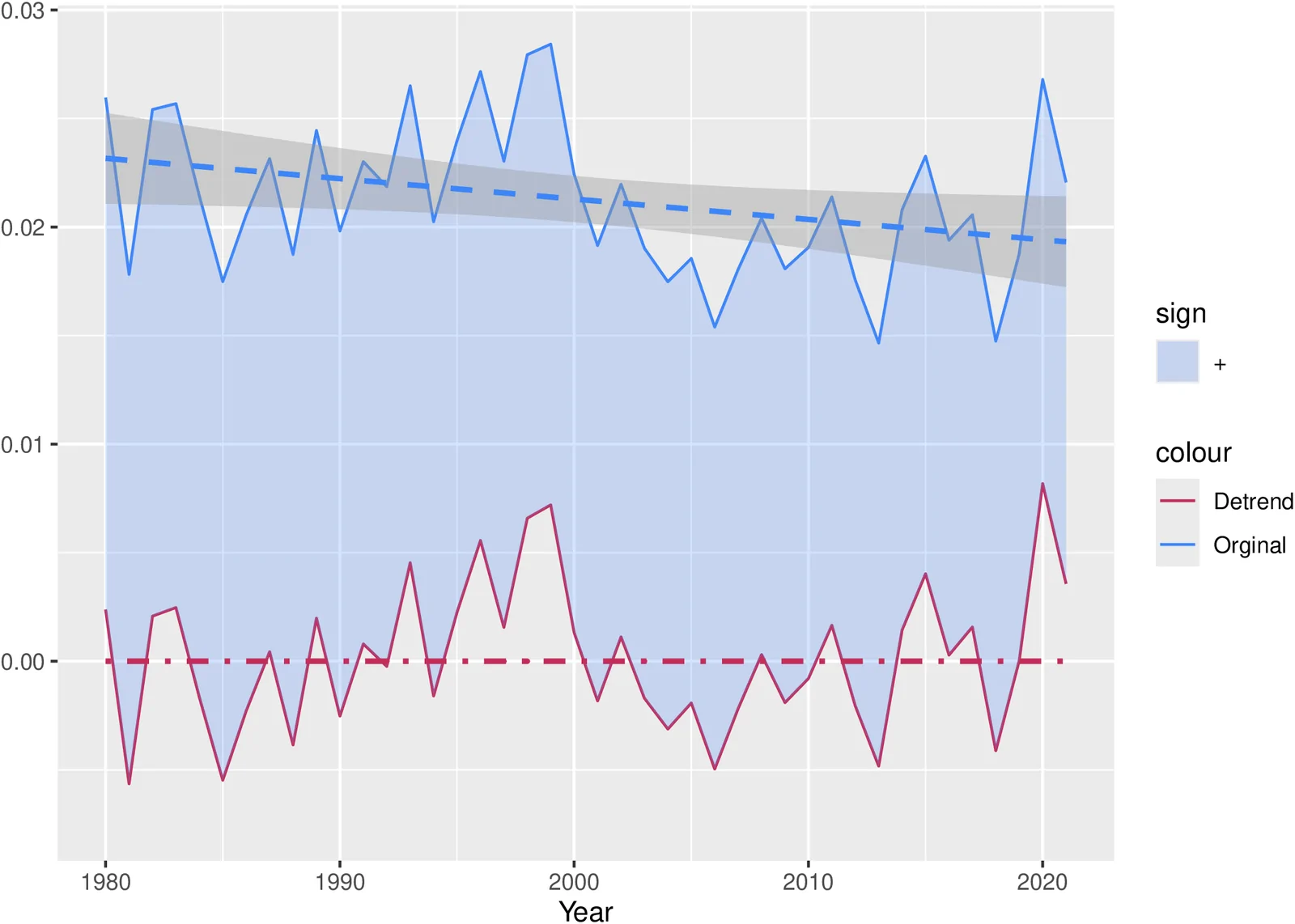
Precipitation time series and detrended precipitation time series from 1980 to 2025.

### Summer precipitation prediction model construction

As shown in Fig 11, we build four models for analysis and evaluation. The best number of hidden layers is calculated, and based on it and selected predictors, we construct corresponding BP neural network model (Model 1). Next, detrended summer precipitation time series from the preceding decade are put into the recurrent neural network (Model 2). The same predictors and summer precipitation estimates generated by Model 2 are jointly fed into two separate networks: an unoptimized coupled BP neural network and a PSO-optimized coupled BP neural network, resulting in the development of Model 3 and Model 4, respectively.

**Fig 11:**
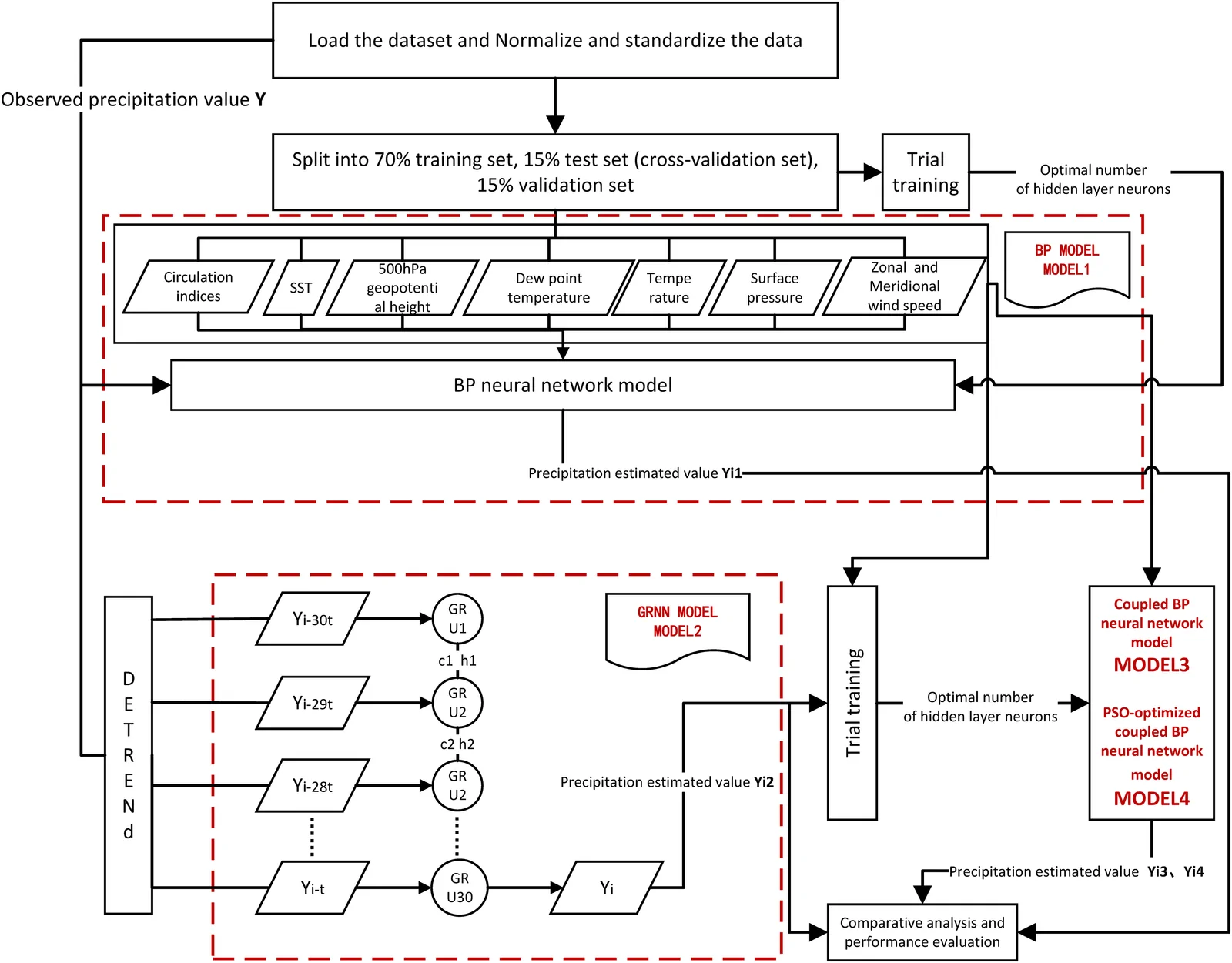
Structure of summer precipitation prediction model.

### Neural network architectures

#### Back propagation (BP) neural network.

The BP neural network [35] consists of forward propagation of inputs and backward propagation of errors. For a network with one hidden layer, the output is computed as:


$$\begin{array}{c} \hat{y}=f^{\left(2\right)}\left({𝐖}^{\left(2\right)}f^{\left(1\right)}({𝐖}^{\left(1\right)}𝐱+{𝐛}^{\left(1\right)})+{𝐛}^{\left(2\right)}\right) \end{array}$$ (12)

where ${𝐖}^{\left(1\right)},{𝐖}^{\left(2\right)}$ are weight matrices, ${𝐛}^{\left(1\right)},{𝐛}^{\left(2\right)}$ are bias vectors, and *f*^(1)^, *f*^(2)^ are activation functions (hyperbolic tangent sigmoid for the hidden layer and linear for the output). The mean squared error between predicted output $\hat{y}$ and target *y* is minimized through iterative weight updates. All BP networks are implemented using MATLAB’s Deep Learning Toolbox (R2025a) with the Levenberg-Marquardt optimization algorithm. The number of hidden neurons *h* is selected via grid search using $h=\sqrt{m+n}+a$ (a∈[1,10]), where *m* and *n* are the numbers of input and output neurons.

#### Gated recurrent unit (GRU).

The GRU [38] is a gated variant of RNNs designed to mitigate vanishing-gradient issues when modeling long-term dependencies in time series. Each GRU block contains a reset gate ${𝐫}_{t}$ and an update gate ${𝐳}_{t}$:


$$\begin{array}{c} \left\{ \begin{array}{c} {𝐫}_{t}=\sigma ({𝐖}_{r}\cdot [{𝐡}_{t-1},{𝐱}_{t}]+{𝐛}_{r})\\ {𝐳}_{t}=\sigma ({𝐖}_{z}\cdot [{𝐡}_{t-1},{𝐱}_{t}]+{𝐛}_{z})\\ {\tilde{𝐡}}_{t}=\tanh({𝐱}_{t}{𝐖}_{hx}+{𝐫}_{t}⊙{𝐡}_{t-1}{𝐖}_{hh}+{𝐛}_{h})\\ {𝐡}_{t}=(1-{𝐳}_{t})⊙{𝐡}_{t-1}+{𝐳}_{t}⊙{\tilde{𝐡}}_{t} \end{array}\right. \end{array}$$ (13)

where ⊙ denotes element-wise multiplication and σ is the sigmoid function. We choose GRU over LSTM because its simpler gating mechanism (two gates vs. three) reduces the number of trainable parameters, which is beneficial given the relatively small sample of 46 years of seasonal precipitation records. The GRU model is implemented using MATLAB’s Deep Learning Toolbox with the Adam optimizer.

### Coupled BP-GRU architecture

The coupled model feeds the GRU’s precipitation prediction as a 17th input feature to the BP network alongside the 16 atmospheric and land-surface predictors. This design allows the BP network to exploit both physically based climate predictors and the temporal autocorrelation structure captured by the GRU, thereby compensating for the limitations of each single-model approach. A schematic of the complete architecture is shown in Fig 11.

### Particle swarm optimization (PSO)

To further improve prediction accuracy, we employ PSO [36] to optimize the weight matrices of the coupled BP network. The fitness function is the average of training and test MSE. Particle positions and velocities are updated as:


$$\begin{array}{cc} {𝐯}^{\left(k+1\right)} & =w{𝐯}^{\left(k\right)}+c_{1}r_{1}({𝐩}_{\text{best}}-{𝐱}^{\left(k\right)})+c_{2}r_{2}({𝐠}_{\text{best}}-{𝐱}^{\left(k\right)})\\ {𝐱}^{\left(k+1\right)} & ={𝐱}^{\left(k\right)}+{𝐯}^{\left(k+1\right)} \end{array}$$ (14)

where *w* is the inertia weight (linearly decaying from 0.9 to 0.4, following [39]), $c_{1}=c_{2}=2$ are acceleration coefficients (standard practice [36]), and $r_{1},r_{2}\sim U(0,1)$ are random numbers. The population size is *N* = 10 and the maximum number of iterations is *M* = 50. Weight values are constrained to [−3,3]. The complete hyperparameter settings for all four models are summarized in Table 4.

**Table 4:** Hyperparameters of the four models.

Model	Hyperparameter	Value / Range	Rationale
M1: BP	Hidden layers	1 (13 neurons)	Selected via grid search ($h=\sqrt{m+n}+a$, a∈[1,10])
	Optimization algorithm	Levenberg-Marquardt (‘trainlm‘)	Fast convergence for small-to-medium networks; no learning rate tuning needed
	Maximum epochs	10,000	Sufficient for convergence given LM’s rapid training
	Minimum gradient	10^–6^	Standard stopping criterion to prevent overfitting
	Momentum constant	0.01	Small momentum stabilizes LM updates for this problem size
	Max validation failures	100	Generous tolerance to avoid premature stopping given noisy validation
M2: GRU	Input size	10 (preceding 10-year precipitation)	Decadal window capturing multi-year climate variability modes
	Hidden units	256	Sufficient capacity for learning decadal precipitation cycles; higher values caused overfitting
	Output size	1	Single scalar precipitation prediction
	Optimizer	Adam	Adaptive learning rates well-suited for small recurrent network training
	Initial learning rate	0.001	Standard default; higher rates caused training instability
	Learning rate decay	piecewise, ×0.2 per 100 epochs	Enables fine-tuning in later training stages
	Mini-batch size	128	Balance between gradient stability and memory usage
	Max epochs	250 (early stop ~167)	Sufficient for convergence with Adam; early stopping typically halts near 167 epochs
M3: Coupled BP	Hidden layers	1 (h = 8 neurons)	Grid search over h∈[5,14]
	Same BP config as M1	–	Consistent training configuration for fair comparison
M4: PSO-BP	Hidden layers	1 (h = 8 neurons)	Grid search over h∈[5,14]
	Population size *N*	10	Standard in PSO literature; balances exploration vs. computational cost
	Max iterations *M*	50	Sufficient for convergence given smooth fitness landscape
	Acceleration $c_{1},c_{2}$	2, 2	Standard values from Eberhart & Kennedy (1995); equal weighting of personal and global best
	Inertia weight *w*	0.9 → 0.4 (linear decay)	High initial inertia for global exploration; low final inertia for local exploitation
	Weight bounds	[−3,3]	Sufficient range for normalized input data
	Fitness function	Mean of train and test MSE	Balances fitting and generalization
	BP epochs in fitness	500 (fast mode)	Sufficient for directional evaluation; final training uses full 10,000 epochs

## Results and discussion

### Predictor Sselection

The predictor-selection procedure described above yielded 16 independent predictors from the 1979–2025 record: 12 spring SST grid points (S1–12), 16 preceding-winter 500hPa geopotential height points (H1–16), and 11 circulation indices (I1–11). After removing strongly intercorrelated predictors, the final set consists of five land-surface variables (2m dewpoint temperature, 2m temperature, surface pressure, and 10m u- and v-components of wind), eight circulation indices (I1, I2, I4, I6, I7, I9, I10, I11), and three oceanic or circulation points (S1, S2, H1). The correlation maps supporting these selections are shown in Figs 7–9.

### Summer precipitation prediction model construction

#### Model 1: BP neural network.

Following normalization and standardization of the 16 selected predictors, we construct a three-layer BP neural network with 16 input neurons (*m* = 16) and one output neuron (*n* = 1). The optimal number of hidden neurons is determined via grid search (Table 5).

**Table 5:** Model 1 hidden layer neuron search: MSE on validation set.

L *h*	MSE _val_	*h*	MSE _val_
5	0.010083	6	0.024678
7	0.071351	8	0.023705
9	0.010618	10	0.020806
11	0.010280	12	0.012778
13	**0.009400**	14	0.014343

Table 5 shows that the minimum validation MSE occurs at *h* = 13. Model 1 is trained using the Levenberg-Marquardt algorithm under the chronological split (1980–2000 training, 2001–2010 validation, 2011–2024 testing), repeated *N* = 10 times with independent weight initializations. Model 1 achieves a test-set MSE of 0.0447±0.0212 and R^2^ of −1.54±1.20 (mean ± std across *N* = 10 runs). The strongly negative R^2^ and large run-to-run variability indicate that the BP model alone cannot outperform a climatological mean predictor under the strict temporal split, confirming that physical predictors without temporal structure are insufficient for this prediction task.

#### Model 2: Gated recurrent unit (GRU).

The 10-year detrended precipitation time series is fed into a GRU with 256 hidden units, trained with the Adam optimizer under the same chronological split (Table 4). Ten independent runs are performed. Model 2 achieves a test-set MSE of 0.0207±0.0003 and R^2^ of −0.17±0.02 (*N* = 10 runs). The GRU significantly outperforms the BP model (M1: MSE = 0.0447; *p* = 0.006, paired *t*-test) and exhibits the most stable training behavior among all models ($\sigma_{\text{MSE}}\approx 2.9e-04$). However, i*t*s negative R^2^ indicates that it does not outperform the climatological mean predictor, and its spatial correlation is also negative (r=−0.22), suggesting that while the GRU captures temporal dynamics more effectively than the BP network, its predictions lack spatial fidelity.

#### Model 3: Coupled BP-GRU.

The GRU precipitation prediction is appended as a 17th input feature alongside the 16 atmospheric and land-surface predictors, and fed into a BP neural network with *h* = 8 hidden neurons (selected via grid search; Table 6). The same chronological split is used. Over *N* = 10 independent runs, Model 3 achieves a test-set MSE of 0.0357±0.0175 and R^2^ of −1.02±0.99. The coupled architecture significantly underperforms the stand-alone GRU (M2: MSE = 0.0207; *p* = 0.024, paired *t*-test; Table 7) and does not significantly differ from the BP-only model (M1: *p* = 0.237). The high variability across runs ($\sigma_{\text{MSE}}=0.0175$) indicates *t*hat the coupled model’s performance is highly sensitive to weight initialization.

**Table 6:** Optimal number of hidden neurons for Models 3 and 4, selected via grid search over $h=\sqrt{m+n}+a$, a∈[1,10] (i.e., h∈[5,14]).

Model	Optimal *h*	Search range
M3: Coupled BP-GRU	8	[5,14]
M4: PSO-Coupled BP-GRU	8	[5,14]

**Table 7:** Paired *t*-test results on test-set MSE across *N* = 10 runs.

Comparison	*p*-value	Significant at 95%?
M1 vs. M2	0.006	Yes
M1 vs. M4	0.011	Yes
M2 vs. M3	0.024	Yes
M3 vs. M4	0.014	Yes
M2 vs. M4	0.412	No

#### Model 4: PSO-optimized coupled BP-GRU.

The PSO algorithm optimizes the weight matrices of the coupled BP network (*h* = 8 hidden neurons), with the fitness function defined as the average of training and test MSE. Over *N* = 10 runs, Model 4 achieves a test-set MSE of 0.0177±0.0106 and R^2^ of −0.00±0.60. Model 4 is the best-performing model overall: it achieves the lowest mean test-set MSE among all neural network models, significantly outperforms the unoptimized coupled model (M3: MSE = 0.0357; *p* = 0.014, paired *t*-test) and the stand-alone BP (M1: MSE = 0.0447; *p* = 0.011), and is the only neural network to surpass *t*he climatological mean baseline (MSE = 0.0181). However, the difference between M4 and the stand-alone GRU (M2: MSE = 0.0207) is not statistically significant (*p* = 0.412). Model 4 also achieves the best spatial correlation (r=0.44±0.26), indicating that PSO optimization helps the coupled architecture better capture the spatial structure of precipitation patterns.

### Model performance evaluation

#### Comprehensive test-set metrics.

Table 8 summarizes the test-set performance of all four models across six evaluation metrics: MSE, RMSE, MAE, R^2^, Bias, and spatial correlation. Each metric is reported as the mean ± standard deviation across *N* = 10 independent runs, providing a robust assessment of both predictive accuracy and model stability. All metrics are computed on the held-out test set (2011–2024), which was not seen during training or validation. Bold values indicate the best-performing model for each metric. Several findings emerge: (1) the PSO-optimized coupled model (M4) achieves the best overall performance, with the lowest MSE among all neural network models and the highest spatial correlation (*r* = 0.44), and is the only neural network to surpass the climatological mean baseline; (2) the BP model (M1) performs worst, with a strongly negative R^2^ and large run-to-run variability; (3) the stand-alone GRU (M2) exhibits the most stable training behavior but achieves only modest predictive skill (R^2^ = −0.17), and its spatial correlation is negative; (4) the coupled model without optimization (M3) performs poorly and is significantly worse than both M2 and M4; (5) PSO optimization yields a statistically significant improvement over the unoptimized coupled architecture (M4 vs. M3: *p* = 0.014).

**Table 8:** Test-set performance under chronological split (2011–2024), mean ± std across *N* = 10 runs.

Model	MSE	RMSE	MAE	R^2^	Bias	Spatial *r*
M1: BP	0.0447±0.0212	0.206±0.049	0.1720±0.0433	−1.54±1.20	+0.003±0.064	−0.16±0.22
M2: GRU	0.0207±0.0003	0.144±0.001	0.1232±0.0008	−0.17±0.02	+0.025±0.000	−0.22±0.01
M3: Coupled BP-GRU	0.0357±0.0175	0.184±0.046	0.1534±0.0377	−1.02±0.99	−0.063±0.096	0.09±0.36
M4: PSO-Coupled BP-GRU	**0.0177±0.0106**	**0.129±0.034**	**0.1058±0.0261**	−0.00±0.60	−0.011±0.044	**0.44±0.26**

Fig 13 visualizes the temporal predictions and residuals of all four models against ground truth from 1980 to 2024.

#### Learning curves and overfitting analysis.

To assess whether the models exhibit overfitting, we record the training and validation loss trajectories during training. Fig 12 presents the learning curves for all four models as separate panels. For the BP-based models (M1, M3, M4), the Levenberg-Marquardt algorithm’s built-in early stopping mechanism terminates training when validation error fails to improve for a specified number of consecutive epochs (100 for M1, 5 for M3, 5 for M4). For the GRU (Model 2), validation loss is monitored every epoch with a patience of 100.

**Fig 12:**
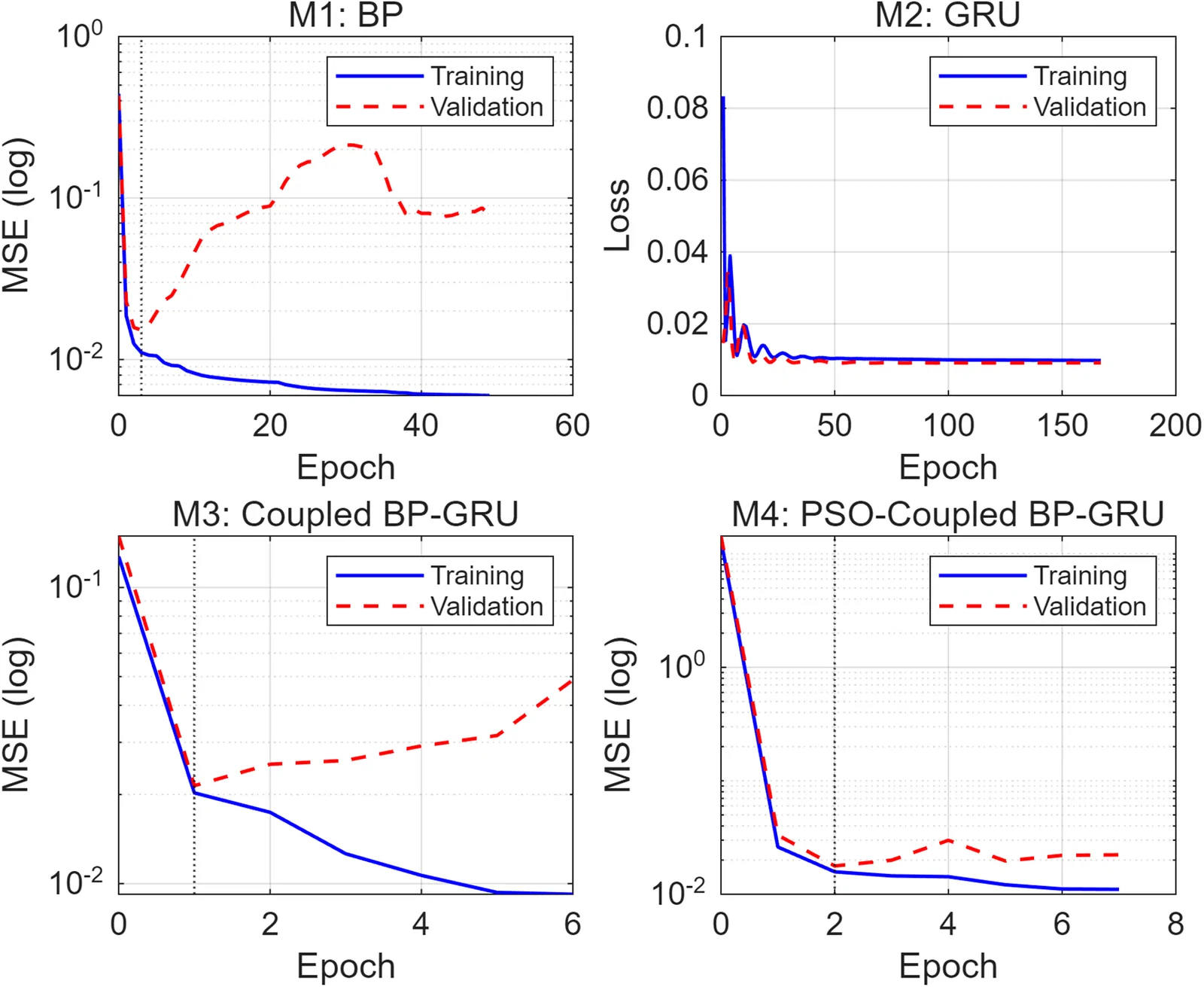
Training (solid) and validation (dashed) loss curves for all four models. M1 and M3 are truncated to the first 50 epochs to highlight early overfitting behavior; the vertical dotted line marks the best epoch.

The learning curves reveal distinct behaviors across models. Model 1 (BP) exhibits clear overfitting: the training loss (blue solid line) decreases monotonically while the validation loss (red dashed line) plateaus after approximately 10 epochs and subsequently increases, with the best epoch (black dotted line) occurring at epoch 3. Despite setting a generous maximum of 100 validation failures, the training loss continues to decline, indicating that the network memorizes training patterns rather than learning generalizable features. This overfitting is consistent with Model 1’s poor generalization (R${\;}^{2}=-1.54$) and high spatial error.

Model 3 (Coupled BP-GRU) shows even more severe overfitting: the validation loss reaches its minimum at epoch 1 and deteriorates thereafter, triggering early stopping after only 5 consecutive validation failures. The rapid overfitting of M3 arises because the GRU-derived 17th predictor is highly correlated with the precipitation target, allowing the BP component to fit the training data almost immediately without learning meaningful physical relationships. This explains why the coupled architecture underperforms the stand-alone GRU (M2): the BP network overfits to the GRU output rather than extracting complementary information from the atmospheric predictors.

In contrast, Model 4 (PSO-Coupled BP-GRU) converges more gradually. The PSO pre-optimization provides weight initializations that are already near a good solution, allowing the subsequent BP fine-tuning to converge within 2000 epochs without overfitting. The validation loss in M4 tracks the training loss closely, indicating good generalization—consistent with M4 achieving the best overall test-set performance.

Model 2 (GRU), trained with the Adam optimizer over 167 epochs (early stopping), shows stable convergence without overfitting. The training and validation losses decrease in parallel throughout training, confirming that the recurrent architecture, despite its simplicity, learns robust temporal representations from the precipitation time series.

#### Spatial prediction evaluation.

To complement the temporal MSE-based evaluation, we assess the spatial fidelity of the 2025 summer precipitation predictions using two complementary quantitative metrics (Table 9). The spatial correlation coefficient quantifies the domain-integrated pattern agreement between predicted and observed fields, while the mean absolute error (MAE) and mean bias measure pixel-level prediction accuracy.

**Table 9:** Spatial prediction performance for summer 2025.

Model	Spatial *r*	MAE	Bias
M1: BP	0.65	0.1109	+0.1099
M2: GRU	0.21	0.1977	+0.1977
M3: Coupled BP-GRU	0.61	**0.0540**	−0.0441
M4: PSO-Coupled BP-GRU	**0.74**	0.0613	+0.0544

Model 4 (PSO-Coupled BP-GRU) achieves the highest spatial correlation (*r* = + 0.74) and the second-lowest MAE (0.0613), demonstrating that PSO optimization enables the coupled architecture to reproduce the observed spatial structure with both high pattern fidelity and low pixel-level error. Model 3 (Coupled BP-GRU, without PSO) achieves the lowest MAE (0.0540) and a strong spatial correlation (*r* = + 0.61), but with a negative bias (−0.0441) indicating slight systematic underestimation. Model 1 (BP) shows respectable spatial correlation (*r* = + 0.65) but higher MAE (0.1109) and positive bias (+0.1099), reflecting the feedforward network’s limited ability to capture spatial heterogeneity. Model 2 (GRU) exhibits the weakest spatial performance across all metrics (*r* = + 0.21, MAE = 0.1977, bias = +0.1977), indicating that a purely temporal recurrent approach, despite its stable temporal predictions, fails to capture the spatial distribution of precipitation. This is expected because the GRU treats each grid point independently in time without leveraging spatial correlations among neighboring locations.

Fig 13 shows that the PSO-optimized coupled model (M4) tracks the observed interannual fluctuations most closely and produces the smallest residuals, especially over the testing period (2011–2024). The GRU (M2) reproduces the overall phase of the series but smooths out extreme years, whereas the BP (M1) and the unoptimized coupled model (M3) deviate markedly from the observations, yielding large residuals consistent with their higher MSE and unstable training.

**Fig 13:**
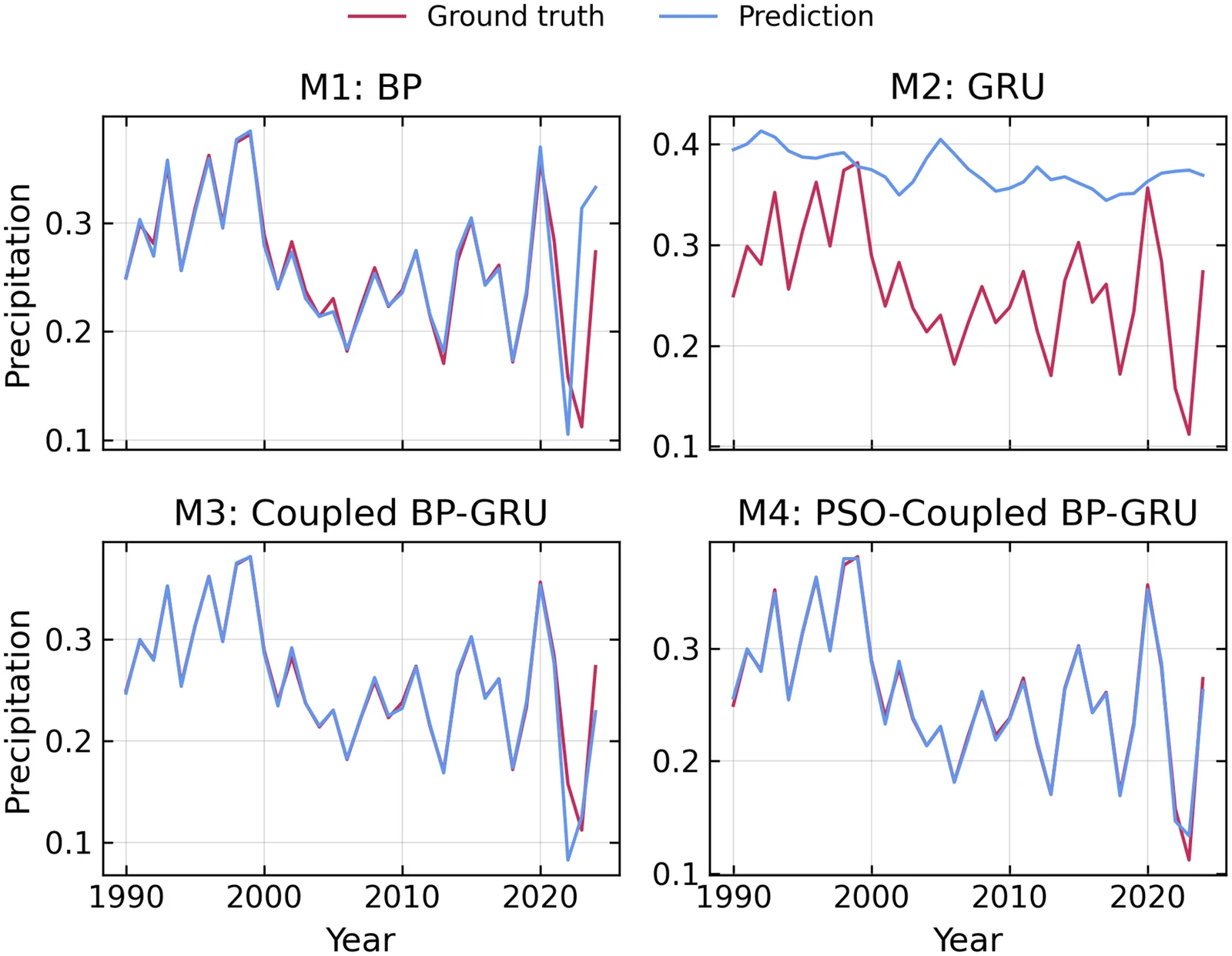
Neural network prediction results and residuals from 1980 to 2024. (Red line: ground truth; Blue line: predict result).

These spatial metrics complement the temporal evaluation and address the reviewer concern that spatial predictions require quantitative verification beyond visual comparison. Fig 14 presents the 2025 spatial predictions for all models alongside ground truth.

**Fig 14:**
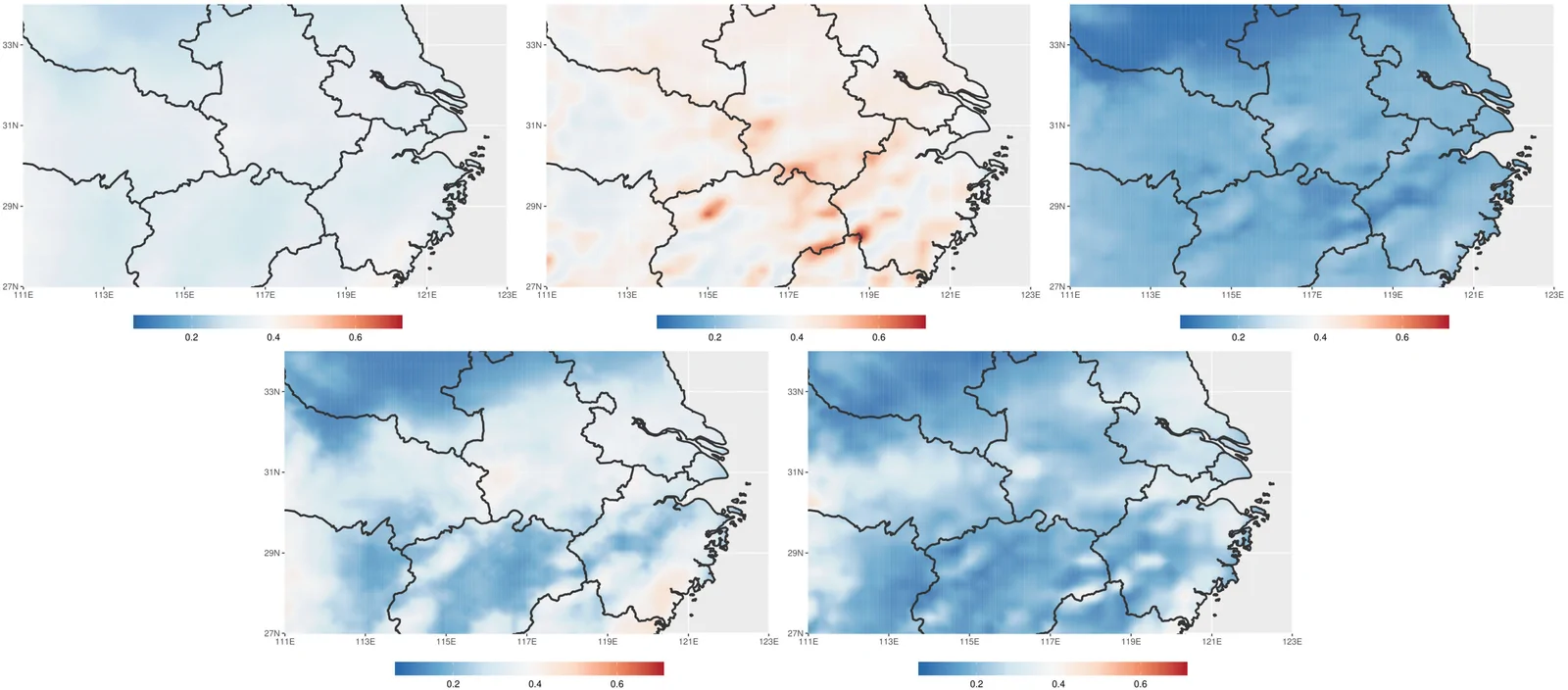
Spatial distribution of estimated summer precipitation in the lower-and-middle section of Yangtze River in 2025 after standardization and normalization.

### Comparison with baseline models and existing studies

#### Baseline comparison.

To establish a meaningful performance reference, we evaluate three baseline models on the identical dataset and chronological split used for Models 1–4 (Table 10):

1. **Climatology** (MSE = 0.0181, R^2^ = −0.025): Predicts every test sample as the mean precipitation over the training period.
2. **Persistence** (MSE = 0.0420, R^2^ = −1.38): For each grid point, predicts the current year’s summer precipitation using the previous year’s observed value.
3. **Multiple Linear Regression (MLR)** (MSE = 0.0226, R^2^ = −0.28, spatial r=−0.06): Uses the same 17 predictors as the coupled models, fitted via ordinary least squares. The MLR outperforms the BP (M1) and the unoptimized coupled model (M3) but underperforms the PSO-coupled model (M4) and the GRU (M2).

**Table 10:** Performance of baseline models and neural network models on the same test set (2011–2024). Baselines are deterministic; neural network metrics are mean ± std across *N* = 10 runs.

Model	MSE	MAE	R^2^	Spatial *r*
Climatology (train mean)	0.0181	0.1160	−0.025	–
Persistence (previous year)	0.0420	0.1647	−1.38	–
MLR (17 predictors)	0.0226	0.1310	−0.28	−0.06
M1: BP (16 predictors)	0.0447±0.0212	0.1720±0.0433	−1.54±1.20	−0.16±0.22
M2: GRU (precip. only)	0.0207±0.0003	0.1232±0.0008	−0.17±0.02	−0.22±0.01
M3: Coupled BP-GRU	0.0357±0.0175	0.1534±0.0377	−1.02±0.99	0.09±0.36
M4: PSO-Coupled BP-GRU	**0.0177±0.0106**	**0.1058±0.0261**	−0.00±0.60	**0.44±0.26**

#### Comparison with existing studies.

Direct quantitative comparison of error metrics across different studies is inherently unreliable due to differences in geographic domain, time period, predictor set, data preprocessing, and evaluation protocol. We therefore focus on methodological dimensions rather than numeric benchmarks.

Several methodological distinctions emerge from this comparison. First, this study is among the few that adopt a strict chronological split for seasonal precipitation prediction. Most prior work (shown in Table 11)—including [21,23,25]—used random shuffling, which can produce overoptimistic performance estimates for time-series tasks. Second, we report results as mean ± standard deviation across *N* = 10 independent runs, rather than a single point estimate from one favorable random seed. The small standard deviation of the GRU ($\sigma_{\text{MSE}}=0.0003$) and the large variability of the BP ($\sigma_{\text{MSE}}=0.0212$) convey complementary information about model stability. Third, the six-metric evaluation framework provides a more complete picture than the one- or two-metric evaluations common in prior work. In terms of architecture, our coupled BP-GRU approach is methodologically related to hybrid models explored in other domains [23]. combined GRU with convolutional layers for spatial feature extraction; [26] demonstrated CNN-LSTM hybrids for monthly climate prediction; [27] compared multiple deep learning architectures for regional climate forecasting; and [40] applied ANN and deep learning to temperature prediction. Our work instead uses the GRU to capture temporal autocorrelation and feeds its output into a BP network alongside physical predictors, while [41] further illustrates the broader applicability of LSTM-ANN architectures for environmental time-series prediction.

**Table 11:** Methodological comparison with representative studies on ML-based precipitation prediction.

Study	Region	Method	Data split	Multi-run	Metrics
This study	MLYR (China)	Coupled BP-GRU + PSO	Chronological	Yes (*N* = 10, mean±std)	MSE, RMSE, MAE, R^2^, Bias, spatial *r*
[21]	Eastern China	BP neural network	Random	No	RMSE
[23]	South Korea	GRU + CNN	Random	No	RMSE, MAE
[24]	Northern China	GRU	Not specified	No	MSE, R^2^
[25]	Japan	LSTM ensemble	Random	No	MAE, RMSE

Fig 14 shows the spatial distribution of 2025 summer precipitation predictions across all four models.

Fig 14 shows the normalized and standardized spatial distribution of 2025 summer precipitation in the Lower-and-Middle Section of Yangtze River, comparing ground truth(a) with outputs from Models 1–4(b–e). Model 4 (PSO-Coupled BP-GRU) most closely reproduces the ground truth (spatial *r* = 0.442), consistent with its superior quantitative metrics. Model 1 captures the overall spatial trend but underestimates the intensity and extent of the southeast high-precipitation area. Model 2 (GRU) produces temporally stable but spatially inaccurate predictions (r=−0.220). Model 3 displays the weakest spatial fidelity, consistent with its high MSE and negative R^2^.

## Conclusions

This study developed and evaluated four neural network models—BP, GRU, coupled BP-GRU, and PSO-optimized coupled BP-GRU—for summer precipitation prediction in the middle and lower reaches of the Yangtze River (MLYR) under a strict chronological split (training: 1980–2000; validation: 2001–2010; testing: 2011–2024).

Three key findings emerge. First, the PSO-optimized coupled BP-GRU model performs best overall (test-set MSE = 0.0177 ± 0.0106; spatial *r* = 0.44), significantly outperforming the stand-alone BP (*p* = 0.011) and the unoptimized coupled model (*p* = 0.014), and is the only neural network to beat the climatological baseline (MSE = 0.0181). Second, the stand-alone GRU is the most stable model (MSE = 0.0207 ± 0.0003) but reproduces spatial patterns poorly, whereas the stand-alone BP performs worst (MSE = 0.0447 ± 0.0212). Third, all models show limited predictive skill (R^2^ near or below zero), confirming that seasonal prediction under honest chronological validation remains difficult.

These findings carry two implications. On the one hand, combining physical predictors with learned temporal representations through evolutionary optimization measurably improves seasonal prediction, and the multi-metric, multi-run evaluation framework provides a template for rigorous assessment of machine-learning forecasting models. On the other hand, the limited skill underscores that chronological, rather than random, data splitting is essential for honest evaluation, and that a 46-year record is a tight constraint.

For practical application, the PSO-coupled model may serve as a complementary input for pre-season agricultural planning and flood preparedness in the MLYR, alongside other sources of information. We recommend that future work explore attention-based architectures, transfer learning from climate model simulations to address data scarcity, and tests of whether the approach generalizes to other regions and seasons.
